# Immunotherapy for Cancer in Kidney Transplant Patients: A Difficult Balance Between Risks and Benefits

**DOI:** 10.3389/ti.2024.13204

**Published:** 2024-11-25

**Authors:** Mónica Bolufer, Jordi Soler, María Molina, Omar Taco, Anna Vila, Manuel Macía

**Affiliations:** ^1^ Department of Nephrology, Hospital Universitario Germans Trias i Pujol, Barcelona, Spain; ^2^ Redes de Investigación Cooperativa Orientadas a Resultados en Salud (RICORS) 2024, Badalona, Spain; ^3^ Department of Nephrology, Hospital Nuestra Señora de Candelaria, Santa Cruz de Tenerife, Spain; ^4^ Redes de Investigación Cooperativa Orientadas a Resultados en Salud (RICORS) 2024, Santa Cruz de Tenerife, Spain

**Keywords:** kidney transplant, cancer, checkpoint inhibitors, immunotherapy, immunosuppresion

## Abstract

Cancer is a major cause of morbidity and mortality in kidney transplant patients. Unfortunately, the use of new anti-cancer therapies such as immune checkpoint inhibitors (ICPIs) in this population has been associated with rejection rates up to 40%, in retrospective studies. The main challenge is to maintain the patient in a delicate immunologic balance in which, while antitumor therapy defeats cancer the graft is safely protected from rejection. Recent clinical trials with ICPI have included kidney transplant recipients (KTRs) and the results advocate for a paradigm shift in the management of basal immunosuppression. This suggests that downward adjustments should be avoided or, even better, that this adjustment should be “dynamic.” This review summarizes the latest scientific evidence available in renal transplantation under ICPI treatment: case series, prospective studies, histopathologic diagnosis, immunosuppression regimens and new biomarkers. This article will provide the latest information in on this specific field, allowing nephrologists to gain valuable knowledge and to be aware of new approaches to immunosuppression management in oncological kidney transplant patients.

## Introduction

Kidney transplant recipients (KTRs) have a significantly higher risk of developing cancer than the general population. This is a major cause of their associated morbidity and mortality [1]. The increased risk of *de novo* and recurrent cancer is multifactorial and has been attributed to immunosuppression, oncogenic viruses and altered T-cell immunity [2, 3]. Chronic kidney disease and cancer are bidirectionally related, as there are some risk factors that promote both pathologies, such as oxidative stress, chronic inflammation, alcohol and tobacco use, viral infections and aging. Impaired renal excretory function could prolong the plasma half-life of some proinflammatory cytokines such as IL-1 beta (interleukin 1 beta), IL-6, and TNF-alpha, and this could be associated with a persistent proinflammatory state in the body [4]. For example, in a Danish study with 5,594 patients who underwent renal biopsy, a higher rate of cancer rate was observed in those who had a specific type of glomerulonephritis, such as minimal change and membranoproliferative [5].

The introduction of new anti-cancer therapies in the treatment of cancer has transformed the field of oncology. These therapies, also known as immunotherapy, are immune checkpoint inhibitor monoclonal antibodies (ICPI): anti-programmed cell death 1 inhibitors (PD1); anti-programmed cell death ligand 1 inhibitors (PDL1) and anti-cytotoxic T-lymphocyte-associated antigen 4 inhibitors (CTLA4). Malignancies are divided into “solid tumors” and “hematologic malignancies,” with ICPIs being largely reserved for solid tumors (invasive and cutaneous). Recently, it has been estimated that 10.5% of all incident malignancies could benefit from receiving ICI treatment, with 49.7% benefiting from this in terms of oncologic response [6]. Unfortunately, these monoclonal antibodies are not commonly used in the KTR population due to a lack of robust evidence regarding their efficacy and safety. Until 2017, KTR were systematically excluded from ICPI clinical trials. The results of some available retrospective clinical trials were disappointing, with an incidence of 42% of kidney rejection among those KTR treated with ICPI [7]. Before expanding the use of ICPI in KTRs, it is essential to identify the factors that predict the risk of rejection and the presumed response rate of the disease.

In this review, we will present the most important published data on the use of ICPI in the renal transplant population and the new immunomodulatory strategies proposed to preserve the effect of cytotoxic T lymphocytes against the tumor while preventing their deleterious effect on the graft.

## Immunophysiopathology and Biomarkers

The immunophysiopathology of immune checkpoint inhibitors (ICPIs) in kidney transplant patients is a complex interaction between the recipient’s immune system, the kidney graft, and the effect of these monoclonal antibodies on the regulation of the immune response. The balance between avoiding potential rejection and the progression of oncologic disease is poorly understood.

Checkpoint regulatory proteins are responsible for imprinting an activating or inhibitory response phenotype on the T cell. CD80/86 (B7-1/B7-2) expressed by the antigen presenting cell (APC) interacts with the CTLA-4 expressed by the T lymphocyte to induce an inhibitory response. Conversely, CD80/86 (APC) interacts with CD28 (T cell) to elicit an activating response. It is not yet known with which receptor on the T cell the PD-L1 receptor of the tumor cell interacts with to exert an activating function. However, we do know that when PD-L1 interacts with PD-1, the effect is suppressive [8]. ICPI monoclonal antibodies block the inhibitory response, thereby favouring T-cell activation.

One of the key issues in both cancer and kidney transplant recipients is the role of exhausted effector T cells. CD8^+^ exhaustion is a consequence of two events: prolonged and persistent exposure to non-self antigens, such as the tumor cell neoantigens or the antigens of a non-identical kidney allograft [9, 10], and lack of CD4^+^ help [11]. Recent studies in kidney transplant patients have shown that long-term immunosuppressive treatment increases the expression of PD-1 but decreases that of PDL-1 and CTLA-4 [12]. Notably, the modulation differs between patients treated with calcineurin inhibitors (CNI) and those treated with mammalian target of rapamycin inhibitors (mTORi), who express more PD-1 and CTLA-4 [13]. The combination of all these factors explains some of the major mechanisms of allograft rejection associated with the use of ICPI. Here are the main factors that may contribute to the rejection process: 1) reactivation of primed alloreactive T cells by PD-1/PD-L1 blockade at the allograft site; 2) activation of the systemic inflammatory response by reactivation of quiescent T cells; 3) the possible development of new T lymphocytes that recognize antigenic specificities on the tumor that are shared by allogeneic peptides of the graft (cross-reaction); and 4) loss of function of T regs [14].

The use of non-invasive biomarkers to predict each patient’s risk of developing graft rejection after initiation of ICPI is of great interest. Plasma donor-derived cell-free DNA (ddcfDNA) levels increase prior to rejection episodes in patients receiving anti-PD-1 treatment [15, 16]. Elevated ddcfDNA during ICPI treatment identified graft rejection 10–15 days earlier than creatinine elevation in two patients in the Schenk-led clinical trial. It is not currently recommended for clinical decision making, but an increase in ddcfDNA may help to monitor these patients more closely. Another biomarker studied in this clinical scenario is the increase in urinary CXCL-10 levels [17]; although the data are very limited, the authors suggest that elevated urinary levels of CXCL-10 prior to nivolumab treatment could predict early allograft rejection. Recently single cell RNA transcriptomics and T cell receptor sequencing have provided important results to understand the role of different CD8-positive T cell subtypes contributing to acute cellular rejection in this scenario [18]. Using pharmacovigilance and multi-omic data, a bivariate regression model of lymphocyte cytosolic protein 1 (LCP1) and adenosine diphosphate-dependent glucokinase (ADPGK) was developed to predict immune-mediated reactions in patients treated with ICPI, including nephritis [19].

## Observational Data

A number of retrospective studies have been conducted to evaluate the use of ICPI in renal transplant recipients [20]. In this multicentre study of 69 patients, 29 experienced rejection and 66% of them (n = 19) required dialysis. In a series of six patients, Venkatachalam et al. reported poor outcomes for renal transplant recipients with metastatic cancer receiving ICPI, describing a high risk of rejection (50%) and poor remission rates (only one patient with melanoma had remission, but after experiencing mixed rejection and a return to dialysis) [21]. Murakami et al. conducted a large multicentre study (n = 69) to evaluate the safety and efficacy of ICPI in kidney transplant recipients with cancer. They found improved cancer outcomes, but a high risk of acute graft rejection (n = 29; 42%). Of these, 14 cases were confirmed by renal biopsy: 7 mixed rejection mediated by T cells and antibodies and 7 pure cellular rejection mediated by T cells. Most of them, 80%, occurred in the first 2 months after the start of ICPI treatment. After targeted treatment based mainly on high-dose corticosteroids and immunoglobulins, 19 patients (65.5%) lost the graft and returned to dialysis [7]. These high rejection rates are similar to data reported in subsequent literature reviews [22–25]. Tsung et al. demonstrated that ICPI, when used with minimized CNI and steroids, is safe and effective for selected patients with advanced cutaneous squamous cell carcinoma [26]. These studies emphasize the complexity and challenges associated with the use of ICPIs in kidney transplant recipients, underscoring the importance of further research to optimize outcomes in this population.

## Interventional Studies

As mentioned above, KTRs have been consistently excluded from clinical trials involving of ICPI due to lack of efficacy concerns and fear of inducing allograft rejection. Recently, this situation has changed with the publication of three prospective, single-arm, phase 1/2 studies in the past 2 years. Currently, another study on the use of ICPI in kidney transplant recipients is registered on clinicaltrials.gov [27].

In 2022, Carroll et al. published the first study on ICPI in KTR. The study population consisted of high immunologic risk patients with stable renal function. The immunosuppressive regimen was not modified prior to the initiation of the ICPI treatment. Only seventeen patients, with either skin or solid tumors, were enrolled (intended to treat) before the early stop of the trial due to the COVID-19 pandemic. The study demonstrated a response to anti-PD1 (nivolumab) comparable to that observed in the general population with a low rate of rejection (11.8%). One rejection episode was successfully treated with anti-rejection therapy.

Secondly, Hanna et al. conducted a phase 1 clinical trial involving 12 kidney transplant recipients (four of whom were second kidney transplant recipients). All participants had cutaneous squamous cell carcinoma [26]. All patients received anti-PD1 treatment (cemiplimab) and an immunosuppressive regimen based on mTOR inhibitors (sirolimus or everolimus, at trough blood levels of 4–6 ng/dL) combined with corticosteroids (40 mg/day gradually tapered to 10 mg/day on day +7). The antiproliferative agent was discontinued at the time of screening. No patient in the study experienced graft rejection or loss during treatment with cemiplimab.

The third study, published in 2024 by Schenk et al., analysed data from eight low-immunologic risk kidney transplant recipients (KTRs) with skin cancer (melanoma, cutaneous squamous cell carcinoma (SCC), or Merkel cell carcinoma) [28]. All received nivolumab and, in case of disease progression, four additional doses of Ipilimumab followed by another course of nivolumab. Immunosuppression was changed to dual therapy with minimized tacrolimus (2–5 ng/mL) and prednisone 5 mg/day. Two patients experienced mixed cellular and humoral rejection and one patient developed cellular rejection. The authors conclude that dual therapy with tacrolimus and prednisone does not protect against graft rejection and may decrease the antitumor response.

All three studies had significant limitations, including small sample sizes, heterogeneity in terms of inclusion criteria, population characteristics, immunosuppression management, tumor types (different types of cutaneous malignancies and solid tumors), ICPI monoclonal antibodies received, previous lines of treatment, outcomes, and in addition, none of them were controlled. A key from these studies is that the ICPI monoclonal antibodies treatment is feasible in kidney transplant recipients, but patients must be carefully selected to achieve good outcomes.

## Management of Immune Checkpoint Inhibition in Transplant Recipients

Currently, there are no clinical guidelines with robust scientific evidence recommending modification of the immunosuppression regimen in KTR prior to treatment with immune checkpoint inhibitors. It is challenging to find the optimal balance between ensuring that the cancer immunotherapy does not counteract the patient’s immunosuppressive therapy and cause graft rejection, while at the same time ensuring that the immunosuppression given does not make the cancer immunotherapy less effective.

### Histopathologic Diagnosis and Rejection Risk Factors

In published cases with biopsy-proven kidney allograft rejection, the typical diagnosis is T-cell mediated rejection, with less frequent mixed T-cell and antibody-mediated rejection [7, 20, 23, 28–30]. In contrast to acute interstitial nephritis, which occurs 14 weeks after initiation of ICPI, the latency in kidney transplantation is much shorter, usually between 22 and 24 days after initiation of anti-cancer treatment [29, 31–34]. There are overlapping histopathological features between related to ICPI T-cell rejection and acute interstitial nephritis. Adam et al. performed an analysis of 725 immune-related genes and found a high degree of similarity between the two entities. They also identified biopsy-based measurement of IFI27 (IFN-alpha inducible protein 27) gene expression as a potential differentiating marker [35]. IFI27 is an immune response gene involved in interferon (IFN) signaling. Its expression is increased in ICPI-TCMR (T cell mediated rejection) and non-immune checkpoint inhibitor-associated TCMR. No differences were observed in the other groups studied (normal, interstitial nephritis secondary to ICPI, interstitial nephritis secondary to other drugs, BK polyomavirus nephropathy and ICPI-associated glomerulonephritis). In consequence, IFI27 could be a potential biomarker to differentiate ICPI-interstitial nephritis from rejection. When ICPI-induced kidney allograft rejection occurs, the response rate to standard treatment is low, with up to 66% graft loss and return to dialysis [7].

A review of the literature shows that the rejection rates are higher in patients treated with anti-PD1/anti-PDL1 than in those treated with anti-CTLA4 [23, 36, 37], in patients receiving low-dose corticosteroids, and in patients with history of previous graft rejection [14]. One explanatory hypothesis is that CTLA-4 acts primarily in secondary lymphoid organs, modulating early T cell activation in lymph nodes. Since the kidney relies more directly on the PD-1/PD-L1 interaction to maintain peripheral tolerance, PD-1/PD-L1 blockade has a more direct impact on breaking this tolerance. On the other hand, PD-1/PD-L1 signaling affects Treg activity in the renal graft microenvironment more than the CTLA4 axis. Conversely, factors associated with lower rejection rates include a longer time between transplantation and cancer diagnosis [23], the use of mTOR inhibitors, the maintenance of at least two immunosuppressive drugs at the time of ICPI initiation [32, 38], and deceased donor kidney transplantation [7]. Longer latency from transplantation to initiation of a new immunomodulatory therapy against cancer may reduce the risk of rejection due to: increased immunologic tolerance of the graft, greater stabilization of the immune microenvironment (increased T-reg and decreased effector T cells), greater potential for some effector T cells to convert to less active memory T cells or even T-reg, and, in general, less dependence on immunosuppression to maintain the graft.

### Adjustment of Maintenance Immunosuppression to Prevent Allograft Rejection in Patients Treated With ICPI

There is no evidence or clinical guidelines recommending adjustment of immunosuppression prior to the initiation of ICPI. As a result, there is considerable variability in the therapeutic approach to these cases, as evidenced by both retrospective studies and clinical trials.

The majority of authors tend to reduce or discontinue maintenance immunosuppression to manage the patient’s immunologic risk, with the aim of improving tumor response [2]. However, there is currently insufficient evidence to support either of the critical decisions they face; which immunosuppressive drug, if any, can be safely discontinued, or what are the target drug levels to be maintained. In one of the largest published multicenter case series of 65 renal transplant patients, only 34.8% were maintained on the same immunosuppressive regimen prior to initiating ICPI. The most common strategy was the combination of two immunosuppressive agents (46% of the patients), such as steroids with CNI or steroids with mTORi. A triple immunosuppressive regimen was used in only 14 cases [7].

In conclusion, some authors propose two different immunosuppressive treatment strategies in this complex scenario:

a) Dynamic steroid regimen; in this approach, the dose of corticosteroids is increased at the beginning of each immunotherapy cycle and then gradually tapered to the usual maintenance dose [39–41].
b) mTORi conversion; is a classic strategy in the management of immunosuppression in solid organ transplantation to prevent tumor growth due to its antitumor effects [42–44]. Despite the published experience, it is difficult to define the specific isolated effect of mTORi conversion because other treatment strategies are used concurrently at the time of cancer diagnosis.

**FIGURE 1:**
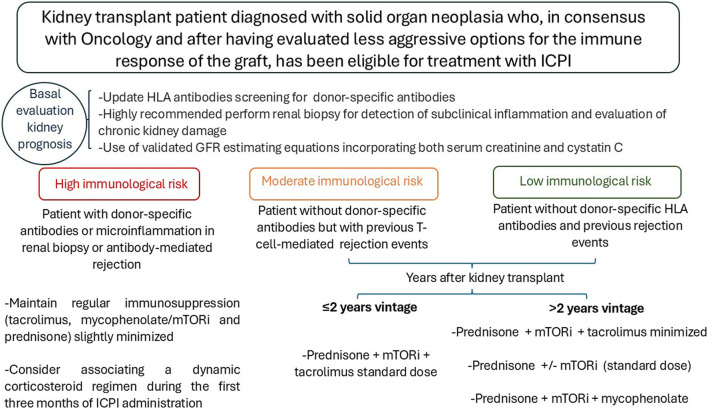
Algorithm for adjusting immunosuppression in kidney transplant patients who will receive ICPI.

As a summary of the available evidence, and always in consensus with the oncology team, the patient and the renal transplant unit, the follow-up algorithm is proposed based on the immunologic risk and time since transplantation. This is a very complex scenario in which multidisciplinary consensus and individualization of each case are essential Figure 1. Recommendations during treatment with ICPI: 1. Check creatinine, urine sediment and proteinuria before each cycle. 2. Avoid the use of proton pump inhibitors, antibiotics, and nonsteroidal anti-inflammatory drugs. 3. Assess for extrarenal immune-mediated manifestations and, if present, monitor renal function more closely. 4. Evaluate monitoring of dd-cf-DNA and/or urinary CXCL-10 levels. 5. Biopsy indicated if rejection is suspected. 6. Balance patient prognosis and renal function prognosis in decision making.

## Discussion

Despite the revolutionary impact of immune checkpoint inhibitors in cancer treatment, there are still significant concerns about their use in kidney transplant patients, given the apparent high risk of organ rejection and the uncertainty about their efficacy in the context of immunosuppression.

However, there is evidence of improved survival in patients receiving triple immunosuppressive therapy. This is particularly true in patients with skin cancer, who actually showed improved outcomes prior to the era of immune checkpoint inhibitors. However, while the results of ICPI therapy appear to be improving in kidney transplant patients, it is important to note that these results remain disappointing in non-skin cancers. Therefore, while we await the results of ongoing clinical trials, the management of cancer treatment in these patients will continue to be highly individualized, particularly in the case of non-skin cancers. The significant risk of graft loss with immune checkpoint inhibitors must be considered. This will be particularly important in patients with a short life expectancy. In such cases, it is important to prioritize the patient’s short-to medium-term outlook and wishes together with the patient’s oncologist. We need to discuss with oncologists the risk of disease progression and death due to the neoplasia if the best available treatment is not received, and carefully weigh this against the options of returning to dialysis in the event of graft rejection. We must not overlook the fact that the loss of a kidney graft can be managed with dialysis by keeping the patient alive.

Cancer is an ongoing challenge in the transplant population. One of the biggest challenges physicians’ faces is the limited opportunity for patients with kidney disease or renal transplant to participate in prospective clinical trials. The establishment of collaborative teams that include both oncologists and nephrologists is a critical step in gradually improving this scarce evidence base. Some hospitals have already established dedicated onco-nephrology units to facilitate the day-to-day management of these patients [45].

Recent data from the first phase I and II clinical trials in transplant patients suggest lower rejection rates than previously published in retrospective studies. However, these results should be interpreted with caution, as the sample size is still very small and cannot be extrapolated to patients with non-cutaneous cancers. Future results from ongoing trials will help to clarify this challenging issue and offer hope to our patients.

## Conclusion

A complex therapeutic strategy is mandatory after the diagnosis of cancer in renal transplant patients and requires a multidisciplinary approach. The risk-benefit ratio of the ICPI in KTRs must be strictly evaluated. Before starting ICPI, it is advisable to maintain at least two immunosuppressive drugs with modulation of the corticosteroid dose and to switch the drug maintenance treatment from CNI to mTORi. Further prospective studies are needed to analyse the risk of rejection in order to predict it with the most accurate and individualized adjustments of immunosuppression.
